# The ecological and biodiversity conservation values of farm dams: A systematic review

**DOI:** 10.1371/journal.pone.0303504

**Published:** 2024-05-13

**Authors:** Michelle Littlefair, Ben C. Scheele, Martin Westgate, David Lindenmayer

**Affiliations:** 1 Sustainable Farms, Fenner School of Environment & Society, The Australian National University, Acton, Australian Capital Territory, Australia; 2 Atlas of Living Australia, Commonwealth Scientific and Industrial Research Organisation, Black Mountain, Canberra, Australian Capital Territory, Australia; Charles University, CZECH REPUBLIC

## Abstract

Biodiversity is in rapid decline globally with agriculture being one of the leading causes. Within agricultural landscapes, some features provide a benefit to biodiversity that is disproportionate to their spatial area. An interesting example is artificial ponds–or farm dams–which can support a large variety of taxa. Here, we present a global review of farm dam research related to biodiversity conservation objectives to provide an overview of the topics, key research insights, and the characteristics of current research. We used a three-stage process to screen literature and identified 104 relevant papers across 27 countries encompassing studies of 13 different taxa. Most of the studies were short-term (less than 5 years) with small sample sizes (less than 20 sites). Of the 104 papers, 88 were focussed primarily on ecological outcomes, such as species richness or abundance, and 15 on primary production outcomes, such as crop and livestock yield, despite addressing or measuring ecological metrics. Only one study measured both ecological and primary production outcomes. Studies frequently examined how the features of dams (79 studies) and attributes of the surrounding landscape (47 studies) impact particular species and communities. Terrestrial mammals (1 study) were under-represented in the literature with macrophytes (28 studies), macroinvertebrates (26 studies), and amphibians (19 studies) receiving the most attention. Our results reveal a growing trend towards recognizing farm dams as habitats for various taxa, including amphibians, beetles, dragonflies, and other macroinvertebrates within agricultural environments. Significant knowledge gaps exist in understanding how dam age, invasive species, and effective management practices impact the biodiversity conservation values of farm dams. Future research should emphasize enhancing biodiversity by collaborating with landholders to increase habitat through strategic vegetation planning, minimizing runoff and nutrient inflow, and restricting stock access.

## 1. Introduction

Major declines in biodiversity are occurring across the globe [1,2] as a result of the intensification of agriculture [3,4], land clearing [3], use of chemicals [5], and the rise in invasive species adapted to modified landscapes [6]. These factors have resulted in substantial declines in groups such as amphibians [7], birds [8] and invertebrates [9]. The persistence of biodiversity in agricultural landscapes is often associated with the presence of features such as farm dams, shelterbelts, rocky outcrops, and large old paddock trees [10–12]. These elements, though frequently of artificial origin, can contribute to biodiversity enhancement in the landscape by offering habitat, promoting heterogeneous landscape structuring, and providing shelter [11].

Farm dams (sometimes also termed ‘ponds’) can serve as valuable assets for biodiversity in many agricultural landscapes [13–16]. We use the term “farm dams” hereafter to distinguish these smaller agricultural reservoirs (~10^2^–10^5^ m^2^) [17] from large-scale irrigation storages and the larger human-made structures used for flood control and/or hydroelectricity. Farm dams often constitute the only wetlands in heavily modified landscapes where natural water bodies have been drained, in-filled and disappeared [17]. Hence, these artificial systems present an opportunity to partly conserve and enhance biodiversity while also contributing to agricultural production [17–20]. While natural wetlands have diminished significantly in number, up to 51% since 1970 in many parts of the world [1], farm dams are abundant with a considerable number of new farm dams being constructed each year [21–23]. For example, there was a 2% per annum increase in the number of farm dams in Australia between 1988 and 2000 and, as of 2019, the continent supported ~ 1.765 million farm dams [17]. In the United Kingdom, approximately 2000 farm dams are created each year [24,25] with the numbers increasing from 425,000 in 1998 to 478,000 in 2007 [24]. The importance of farm dams for conservation is underscored by their capacity to offer distinctive habitat for various taxa. For instance, farm dams support waterbirds through diet provision, enhance the habitat for aquatic macroinvertebrates, and provide breeding habitat for amphibians [26–28]. Recognizing the links between aquatic and terrestrial ecosystems, farm dams have the potential to positively influence not only alpha diversity but also contribute to beta and gamma diversity, further emphasizing their contribution to biodiversity conservation.

The characteristics of farm dams and how they are managed may have important impacts on their value for biodiversity [10,24], with regionally varying benefits [17]. An understanding of the cultural, social, and economic significance of dams in different regions is important to understand the motivation behind, and application of, management practices and help guide conservation plans and outcomes. For example, it is common for dams in North America to be stocked with large numbers of fish for recreational fishing purposes, and for dams in Australia to be used as the primary water source for livestock and irrigation, much like their European counterparts [29,30]. Additionally, in countries with developing agriculture, such as South Africa, farm dams are relied upon as a dependable water source during periods of drought or water scarcity [31]. In each case, these dual purpose/usages could negatively impact biodiversity values. Furthermore, while farm dam configuration varies, they are often designed and constructed to have steep banks and deep basins which promote water retention [17,32] and make it harder for cattle to become bogged in muddy areas [16,32]. Whilst farm dams constructed in this manner provide a reliable source of water for stock and irrigation [21,33,34], they lack shallow edges and littoral zones with vegetation, both characteristics of many natural water bodies [35]. Littoral zones are particularly important for biodiversity as the vegetation provides habitat for foraging, refuge, and egg-laying for organisms such as amphibians, fish, and macroinvertebrates [16,36]. Gentler slopes along the edges of artificial farm dams serve to mitigate the risk of these environments becoming ecological traps, where animals entering the dam are at risk of being unable to exit [37].

Despite being recognized as important for biodiversity, there are still key knowledge gaps about the ecological value of farm dams in agricultural areas. These gaps may limit the development of effective policies, programs, and practical management prescriptions to improve the conservation value of these small water bodies. To date, there has been no global review of the state of the scientific literature focussed specifically on the use of farm dams and the relationships between their structure, condition, management, and their value for biodiversity conservation. The development of an effective conservation strategy for human-made habitats requires the examination of appropriate sampling methodologies, study duration and size, taxonomic group coverage, publication year and the country in which the study was conducted. Studies with longer durations can provide insight into the effects of ecological dynamics over time while a larger sample size may provide more statistically robust results [38,39]. The inclusion of a wide range of taxonomic groups in a study allows for a more comprehensive understanding of ecological dynamics [39]. Publication year is a potential indicator of the most recent developments in the field and whether the research takes those into account [40]. Knowledge about the country where the study was conducted can illuminate the framework of local culture, social, economic and political factors which may influence the local conservation strategies. It is important to consider these factors to develop more informed and effective conservation strategies to address the challenges facing conservation in human-made habitats.

This study aims to critically examine research on farm dams to identify themes that may assist in the development of policies and programs in support of biodiversity. In conducting this review, our goals were to: quantify the literature investigating biodiversity in farm dams, highlight the major knowledge gaps in biodiversity in farm dams, and identify possible avenues to overcome these gaps. We asked the following questions to guide our review:

1. What are the characteristics of conservation-focused research on farm dams (e.g., sample size, study duration, taxonomic group coverage, publication year, and country of study)?
2. Which taxonomic groups are studied, and which biodiversity metrics are used to quantify these groups at farm dams?
3. What are the ecological variables used to predict the biodiversity values of farm dams (e.g., vegetation cover, and water quality)?

## 2. Methods

### 2.1 Multi-stage process–empirical review

**Fig 1:**
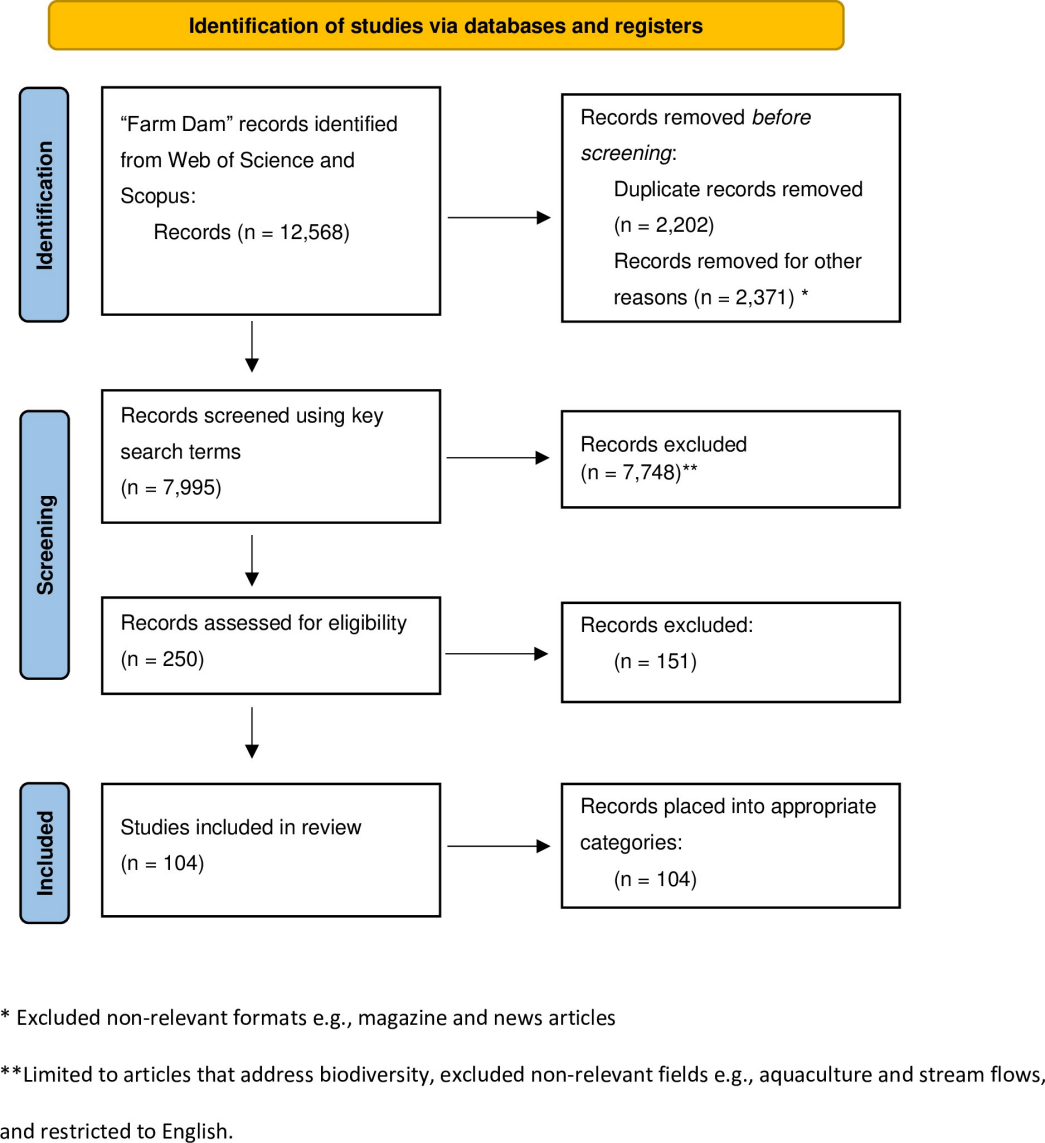
Selection process of included publications from the Clarivate Web of Science and SCOPUS.

Adhering to PRISMA (Preferred Reporting Items for Systematic Reviews and Meta-Analyses) guidelines, we conducted a systematic review of the farm dam literature using a three-stage process (Fig 1). The first stage involved using the databases “Clarivate Web of Science” and “Scopus” to search for peer-reviewed publications that measured ecological variables on farm dams. In each database, we adapted the search to account for syntax differences and include any words shown in Table 1 representing a farm-related context (e.g., TITLE-ABS-KEYWORD (farm* OR agriculture*) with the words shown in Table 1 representing a waterbody (e.g., TITLE-ABS-KEY (dam* OR pond* OR tank*). The two search engines grouped the articles into research areas, enabling us to exclude articles from fields which were not relevant for the purpose of our study, such as aquaculture and hydropower. Studies with an ecological objective that addressed agricultural production, water quality, vegetation or biodiversity were retained. The search was run on September 19^th^, 2023, and identified a total of 7,995 papers that met our search criteria.

**Table 1:** Included and excluded terms for searching farm dam articles, categorized into farm-related and waterbody-related terms.

Included and excluded search terms for further assessment of farm dam articles		
Farm-related	Waterbody-related	Excluded terms
agricultur* agro-*, arable, farm*, non-urban	artificial, cattle ponds, dam* earth bank, earth tank, excavated tank, human-made, human-modified manmade, man-made, pond* reservoir*, waterbod*, water storage, wetland*	arch dam, beaver dam, buttress dam, coffer dam, constructed wetland, diversion dam, embankment dam, gravity dam, hydropower dam, industrial waste dam, levee, masonry dam, overflow dam, regulating dam, saddle dam or dike

The search was adapted to account for syntax differences

(*) and quotation marks were used for phrase searching. Terms applied to titles, abstracts, or keywords.

For the second stage of the review, two independent researchers, distinct from the team of co-authors, alongside the author, screened the titles and abstracts of 7,995 articles with the R statistical language program “revtools” [41], identifying articles focused on biodiversity objectives in farm dams (Table 2).

**Table 2:** The included terms used in part 2 of the screening process to search for appropriate farm dam articles.

Included search terms for the second stage of the assessment of farm dam articles		
abund*, availability, characteristic*, connect* distribut*, ecology*, fauna, habitat*, landscape, occup* organism, presence, reproduce*, selection, use	activit*, biodivers*, communit*, conserve*, divers* endanger*, flora, hotspot, life, occur*, population, quality, restore*, species, vulnerable	assemblage, biology* compos*, detect*, eco-, ecosystem, environment*, function, invasive, manag*, organic, predator*, rar*, rich*, threaten*, wild*

The search was adapted to account for syntax differences

(*) and included prefixes and suffixes e.g., “meta-population” and “populations”, and compound words e.g., “wildlife”. Terms applied to titles and abstracts.

To be included in the subsequent review, each article had to meet the following criteria:

1. Include any of the words shown in Table 1 representing a farm-related context e.g., ‘farm’ or ‘agriculture’ followed directly by any of the words shown in Table 1 representing a waterbody e.g., ‘dam’, ‘tank’, ‘reservoir’, ‘pond’, in the title, keywords or abstract.
2. Include an objective focusing on biodiversity outcomes by using words related with ecology or biodiversity, in the title, keywords or abstract (Table 2).

We excluded grey literature, laboratory studies and all studies pertaining to hydropower dams, industrial waste dams, constructed wetlands and levee, resulting in 250 articles remaining for review.

A full text review was conducted for the previously selected 250 articles to identify those with biodiversity objectives and/or outcomes, and centred around our definition of farm dams, being small (~10^2^–10^5^ m^2^), typically human-made reservoirs used as a water source for crops, livestock, and other agricultural purposes. Studies that did not have or measure a biodiversity relevant outcome, goal or aim were removed (e.g., studies that looked at farm dams as a source of water for human consumption or measured greenhouse gas emissions). This produced 104 articles that fit our selection criteria (S2 Appendix). To identify research patterns within the literature, we extracted the following information from 104 papers: 1) the year of publication, the duration of the study, and the precise location (latitude/longitude or region), (2) the number of farm dams in the study and the target taxon (if any), and (3) the defined research goals or objectives. To ensure meaningful conclusions, this review focused only on research which fulfilled the aforementioned criteria.

#### 2.1.1 Bias assessment

We performed a comprehensive bias assessment focusing on ecological fallacy, confounding variables, data validity and reliability, temporal issues, and geographical boundary generalizations. The goal of this assessment was to critically assess the potential sources of bias and limitations across the 104 papers selected for inclusion. Each of the included papers underwent a thorough bias assessment using the standardised scoring system provided by the Cochrane Collaboration’s Risk of Bias Tool [42]. This resulted in each study being assigned an overall result ranging from low to high. Notably, for a paper to receive a "high" overall ranking, it is judged to be at high risk of bias in at least one domain, or there may be concerns across multiple domains that substantially lowers confidence in the result.

### 2.2 Research questions

#### 2.2.1 What are the characteristics of conservation-focused research on farm dams (e.g., sample size, study duration, taxonomic group coverage, publication year, and country of study)?

We used data collected during the third stage of the review to identify trends in the farm dam literature. We created five categories (“Study Type”, “Taxon”, “Predictor Variable”, “Response Variable”, and “Location”). For example, the category “Study Type” was broken into groups such as “Observational”, “Interventional”, “Long-term”, “Short-term”, and each article was accordingly assigned one or more of these labels (S1 Appendix). Predictor and response variable categories were comprised of a combination of similar variables measured in each paper e.g., the “Dam characteristics” category included variables related to dam configuration such as “Construction design”, “Construction year” and, “Surface area”.

We used the R package ggplot2 [43] to create a heatmap of the data, i.e., a matrix where each entry shows a colour corresponding to the prevalence of articles in a given category. Each heatmap was then used to visualise variation across multiple combinations of two of the data categories [Study Type, Response Variable, Predictor Variable, Taxon] to display patterns of associations.

All 104 articles were evaluated for: 1) duration of study, 2) number of waterbodies, 3) targeted taxonomic groups, and 4) the nature of the sampling methodology, distinguishing between observational and interventional methods. For the examination of global farm dam research over time, survey dates and region were extracted for each study at the level of detail provided by the study and then categorized into year, country, and continent.

#### 2.2.2 Which taxonomic groups are studied and which biodiversity metrics are used to quantify these groups at farm dams?

We conducted a detailed evaluation of farm dam papers with biodiversity objectives (n = 104) to identify variables influencing patterns of biodiversity. We read each paper and recorded the ecological response variables measured, for example species richness. Biodiversity can be classified and measured in several ways depending on the landscape, ecosystem, or habitat subject to evaluation [44–47]. We identified and recorded the taxonomic groups and biota examined in each article, encompassing a range from amphibians, birds, and macroinvertebrates to include invasive and predatory species such as fish and crayfish. Articles that focused on more than one group were recorded across all relevant taxa. We also identified and recorded the “biodiversity metric” (e.g., species richness, abundance, and occurrence) used to measure the biota studied. Heatmaps were produced to visualise patterns of association between the groups of biota that articles examined, and the biodiversity metrics used to quantify those groups.

#### 2.2.3 What are the ecological variables used to predict the biodiversity values of farm dams (e.g., vegetation cover, and water quality)?

Studies assessing farm dams’ biodiversity success were categorized based on their objectives. Studies that included production objectives, along with biodiversity objectives, or examined production objectives in response to biodiversity relevant variables were included. We focused on three target groups: fauna, vegetation, and water quality. This component of our review was descriptive as there were too few samples in each class to make quantitative comparisons. Heatmaps were produced to visualise patterns of association between these variables and the objective of interest. Our approach is a standard method to summarize scientific information where inconsistent objectives and definitions are used, and data are too sparse to allow formal meta-analysis [48].

## 3. Results

We identified 104 relevant articles that examined the biodiversity values of farm dams. The articles came from 66 different publication outlets, with all but two being published in peer-reviewed journals, the remaining studies were theses. Studies were conducted across 27 countries, with the most commonly represented countries being Spain (10%), The United States of America (9%), Japan (6%), Australia (5%), and South Korea (5%).

**Fig 2:**
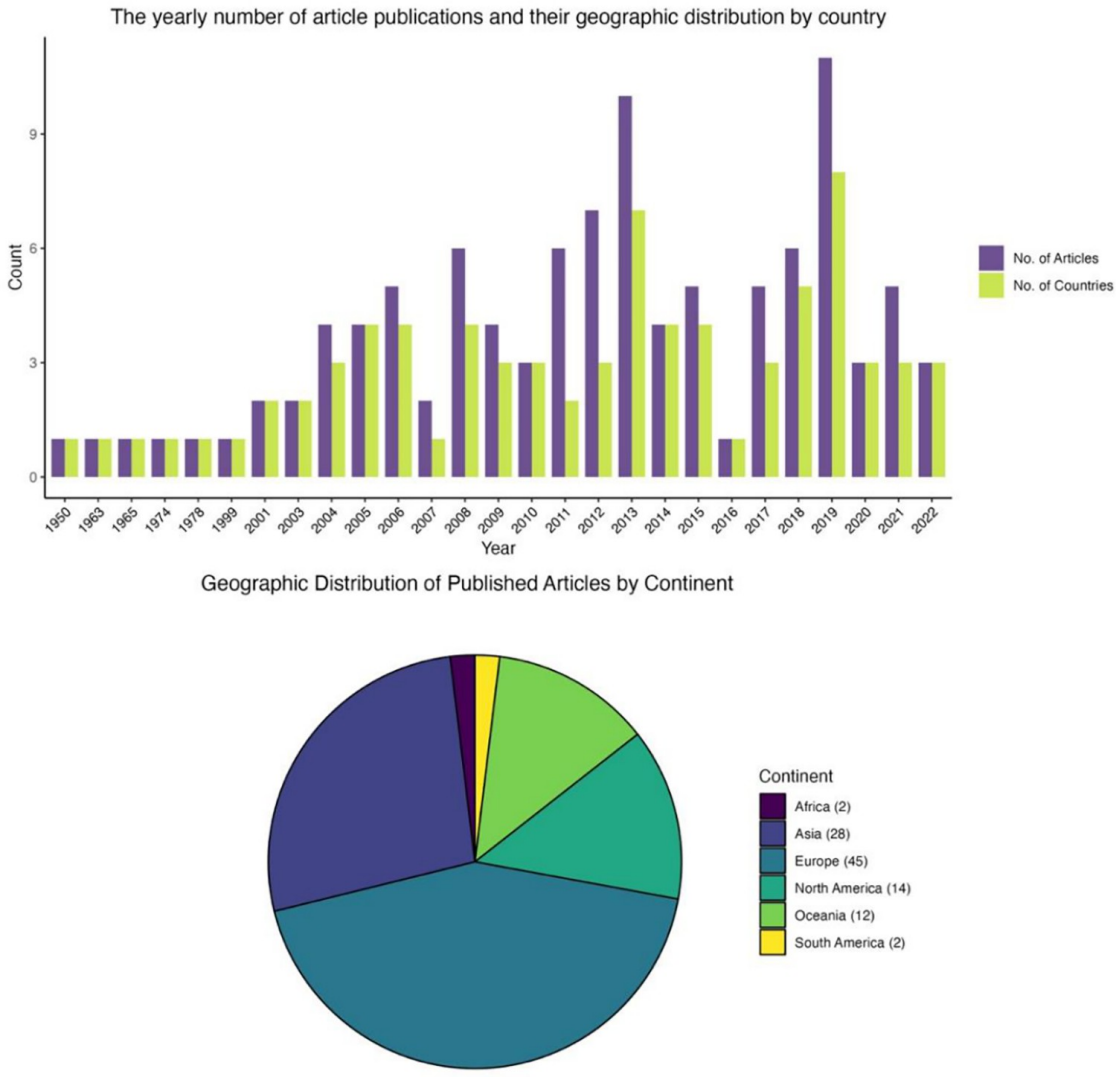
Paired bar and pie charts. The bar chart depicts the number of articles and the number of countries in which these articles were published, sorted by year. The pie chart depicts the total number of published articles, with the count represented in brackets, to increase readability.

We found there has been an increase in the number of studies examining biodiversity in farm dams since 1950 with at least one study per year from 2003. Since 2003, the number of articles published annually has fluctuated between eleven (11) in 2019 and one (1) in 2016 (Fig 2).

### 3.1 Risk of bias assessment

**Fig 3:**
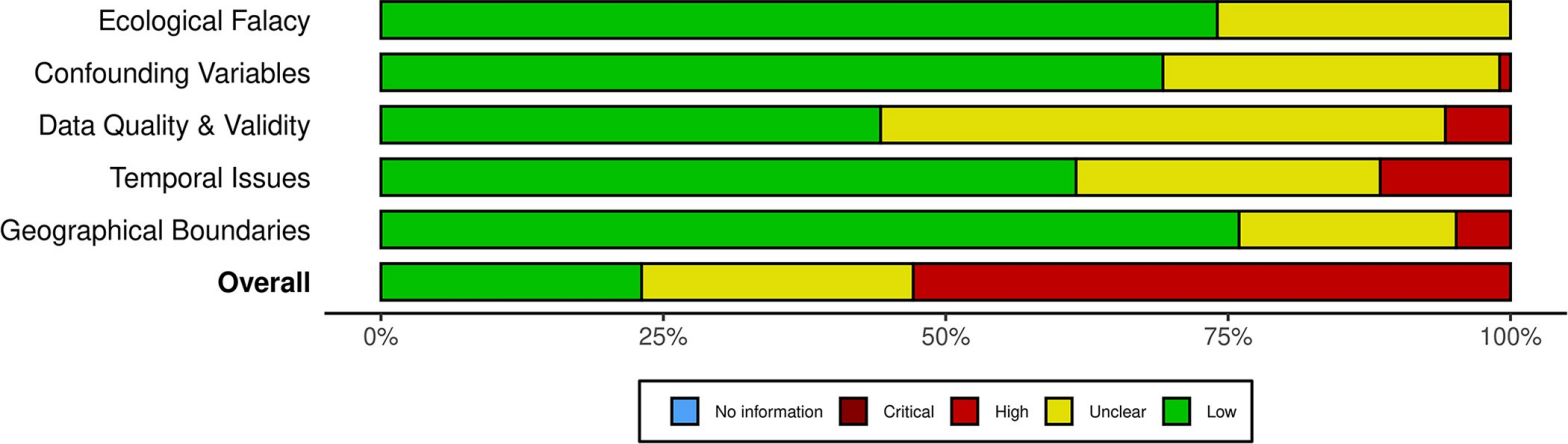
Representative summary of risk of bias across the studies. The leftmost colour, indicated by green, represents a low risk of bias. In the middle, represented by yellow, indicates an unknown risk of bias. Lastly, the rightmost colour, depicted as red, suggests a potentially high risk of bias.

Our risk of bias assessment revealed considerable variation amongst the 104 articles (see S3 Appendix). Studies with an overall ranking of “low” (i.e., low risk for all assessment criteria) made up 23% of the studies, and studies with an overall ranking of “some concern” (i.e., some concern was noted for only one assessment criteria) made up 24%. We found 32% of studies raised some concern in at least two of the assessment criteria, and 20% of studies had a high risk of bias in at least one assessment criteria. This resulted in 53% of studies with an overall ranking of “high” (Fig 3). This commonly resulted from a lack of sufficient detail to rule out the concern for the risk of bias. No studies had a critical risk of bias.

### 3.2 What are the characteristics of conservation-focused research on farm dams (e.g., sample size, study duration, taxonomic group coverage, publication year and country of study)?

The duration of the studies in 97 of the 104 articles ranged from one day up to five years, with the remaining seven articles fitting our definition of long term, exceeding five years or more. The average farm dam study occurred over approximately 18 months. Sample size (number of waterbodies) ranged from two dams to 16,543 dams with a median sample size of 40 and a mean of 345. Ninety-eight studies utilized a sampling methodology that was observational where researchers undertook their investigation without manipulation of the study area or subject, with the remaining six studies using interventions such as building fences around the dams.

Many of the articles had research objectives, aims and goals that were exclusively ecologically and/or biodiversity motivated, commonly answering questions that sought to improve the biodiversity in or around farm dams (n = 75). The remaining studies (n = 29) posed questions that examined agricultural production in response to biodiversity-relevant variables hereafter referred to as production-based studies. Only one study of the 104 examined had aims and goals that addressed both ecological and agricultural outcomes.

### 3.3 Which taxonomic groups are studied and which biodiversity metrics are used to quantify these groups at farm dams?

**Fig 4:**
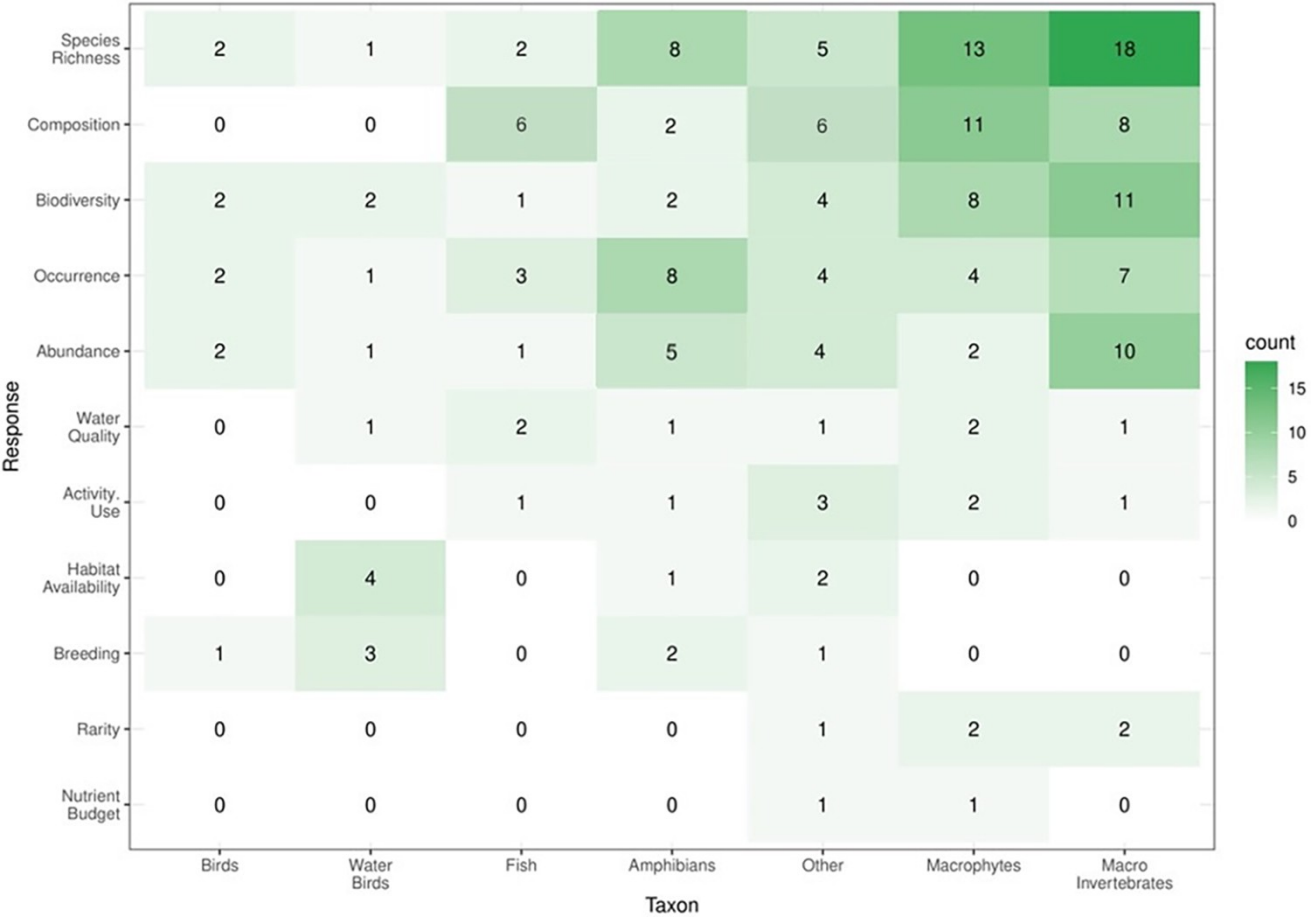
Heatmap showing the taxon studied in each article and the response variable used. The darker colours represent a higher number of articles. Each paper may be represented in more than one category. Category definitions can be found in S1 Appendix.

Of the three target groups that we investigated (fauna, vegetation, and water quality), fauna was the most examined (n = 64) followed by vegetation (n = 32), and water quality (n = 22). Most articles focused on only one of the three categories, while eight described both vegetation and fauna, five described water quality and fauna, two described both vegetation and water quality, and another two described all three of the categories. Macroinvertebrates were the most commonly (n = 28) studied taxonomic group, followed by macrophytes (n = 26) and amphibians (n = 18). Many of the studies used different metrics as proxies to measure biodiversity, with the most common measure being species richness (n = 35) followed by species abundance (n = 18). Species richness was most commonly measured as a response to the physical characteristics of dams (n = 72) and the physical characteristics of the landscape surrounding the dams (n = 43). Of the studies measuring species richness, 18 focused on macroinvertebrates and 13 focused on macrophytes (Fig 4).

### 3.4 What are the ecological variables used to predict the biodiversity values of farm dams (e.g., vegetation cover, and water quality)?

**Fig 5:**
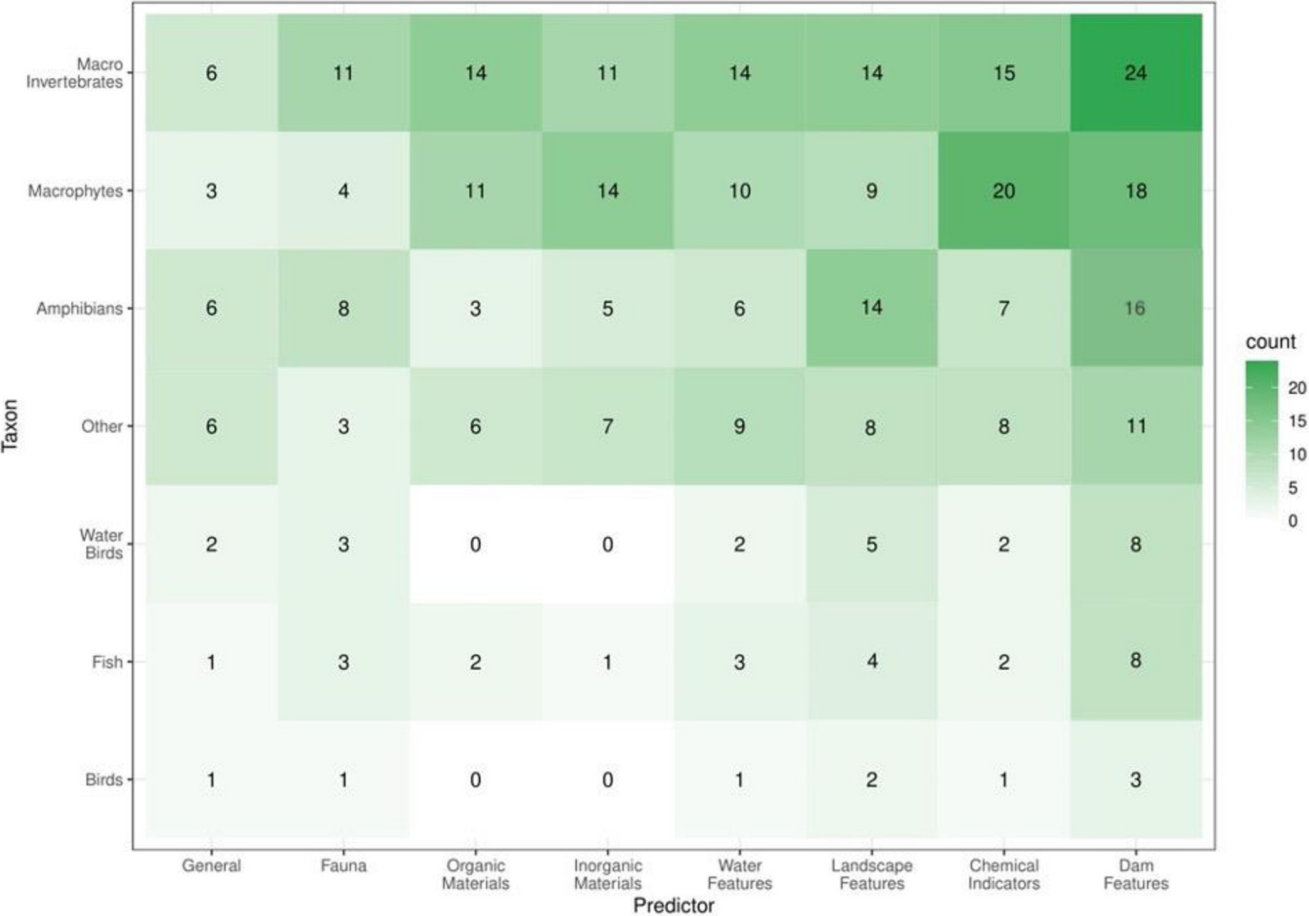
Heatmap showing the taxon studied and the predictor variable used. The darker colours represent a higher number of articles. Each paper may be represented in more than one category. Category definitions can be found in S1 Appendix.

In many of the selected studies, authors considered physical characteristics of dams, such as surface area and vegetation cover, as response variables. Out of those investigations, 48 looked at dam size while 30 explored dam depth, and 26 examined vegetation cover. Further, 24 of the aforementioned studies focused on macroinvertebrates, 18 on macrophytes, and 16 on amphibians (Fig 5).

Vegetation, of great importance in regard to habitat provision and sediment filtration, contributed to the biodiversity value in 14 studies, with the presence and cover of vegetation at dams commonly being studied in conjunction with water birds, (4), amphibians (4), macroinvertebrates (3), and water quality (2).

Six ecological studies explored the influence of water quality on the biodiversity value as well as the factors affecting water quality in farm dams. Eight studies found surrounding land use had the biggest influence on water quality with cattle and chemical run off decreasing water quality. Three studies found the characteristics of the surrounding landscape influenced water quality. Two of these studies showed that the presence of surrounding, fringing and submerged macrophytes improved water quality while the other study found the presence of overhanging woody vegetation (i.e., shade trees and shrubs) and an increased influx of sediment from the surrounding paddocks decreased water quality.

Of the 29 studies with objectives that included production outcomes, 14 examined water quality, with eight finding intensive land use, such as grazing and cropping, had a negative effect on the water quality of dams. Four found the presence of vegetation in dams improved water quality. Two studies looked at water quality as a predictor of biodiversity and found that dams with a higher quality of water (i.e., less heavy metals, nitrogen and phosphorus) were positively correlated with an increase in macrophyte species richness.

## 4. Discussion

We reviewed 104 articles examining biodiversity values of farm dams. Our systematic review highlighted the attention given by researchers to the study of farm dams as potential habitats for various aquatic species. Additionally, our findings indicate a notable focus among researchers on exploring the impact of physical characteristics and management practices of dams on biodiversity. We found species richness and abundance to be among the frequently used response variables across ecological studies, with water quality and vegetation being commonly used predictor variables. Our risk of bias assessment also revealed a noteworthy proportion of studies exhibiting a "high" risk of bias. This highlights the need for improved methodological rigor and transparency in future research to ensure reliable evidence for effective conservation strategies.Ecological studies vary in length depending on the research question, the study design and the duration of the ecological phenomena being studied. This has resulted in a body of literature ranging from a few days to many decades. Most of the studies within our review collected data over periods of less than two years. These short-term studies are important for providing snapshot results; however, long-term studies provide valuable insights into the dynamics and processes of ecosystems over extended periods of time, identifying patterns, trends, and fluctuations that may not be apparent in short term studies to be identified [49]. More funding for long-term ecological studies is required to sustain long-term data collection, equipment, and personnel costs, provide access to new advances in technology, prepare for unforeseen disruptions and leverage the importance of long-term data in conservation and management decisions [50].

We identified trends in the geographic and temporal distribution of research on farm dams. For example, there is a growing focus on understanding the ecological and conservation implications of farm dams, with the number of studies on the biodiversity values of farm dams increasing significantly in recent years. However, the location of studies revealed that most research has been conducted in more affluent regions, such as Europe (n = 45), potentially resulting in a biased understanding of the global ecological importance of farm dams. Furthermore, the research highlights unique regional conservation and management challenges, such as the presence of non-native species [51], construction configuration and different hydrological regimes [52]. This information can not only highlight regions that require additional research efforts but identify opportunities for collaboration between conservation research groups in these different regions.

Integration of production and biodiversity conservation is increasingly recommended as a management approach to improve the sustainability of agricultural systems [53]. However, we found only a small number of studies examined how agricultural outcomes (e.g., livestock use) were influenced by the physical characteristics of dams, such as the influence of vegetation cover on water quality. Furthermore, only one article, a review [54], considered both conservation and production outcomes. The limited number of studies considering both biodiversity and production values of farm dams highlights the need for more research across disciplines and the need to engage landholders to co-design and test interventions that enhance both production and biodiversity values. One way to address this shortcoming could be to prioritize funding schemes that emphasize collaboration between ecological and agricultural researchers as well as landholders and managers.

Ecological research on farm dams has predominantly focused on the physical characteristics of dams and the surrounding landscape. Vegetation and water quality can be relatively easily measured, manipulated, and recorded and are widely known to contribute to biodiversity. Larger dam size and higher vegetation cover and complexity are important predictors of biodiversity, while water quality is crucial for both biodiversity and production outcomes [55]. However, highly vegetated dams are sometimes perceived as a hindrance to agricultural production [16] and improving water quality can involve trade-offs such as increased labour and potential water loss from evaporation through changes in the chemical composition and surface tension of water [56,57].

While the physical characteristics of farm dams are important predictors of biodiversity, it is also essential to consider the ecological needs and preferences of different taxa. Much of the research focused on macroinvertebrates, macrophytes, and amphibians [58,59] with some populations now heavily reliant on farm dams for their persistence [13,60], while studies focusing on mammals were completely absent. This focus could be because farm dams may not be important habitats for many mammal species, and natural habitats, such as wetlands, may be more suitable [51]. Additionally, many of the focal taxa (e.g., amphibians and reptiles), have very specific habitat requirements, are small and have limited dispersal capacity. Therefore, a more holistic approach that considers both the physical characteristics of farm dams and the ecological needs of different taxa is necessary for effective management and conservation of farm dams.

### 4.1. What is the role of farm dams in conservation?

Our systematic review highlighted a prevalent focus on conservation within the research examined. Newer trends in farm dam research suggest a notable transition towards a conservation-centric approach. Studies are increasingly focusing on the role of farm dams in habitat connectivity between naturally occurring waterbodies, the migration of animals across farmland, and reducing the risk of local extinction [12,15,16]. Many articles identified strong relationships between variables such as surface area, vegetation cover and water quality and the occurrence of one or more taxonomic groups, suggesting management strategies can be implemented to improve habitat value and thus biodiversity, especially when management is done in conjunction with effective conservation of existing wetlands [61]. Several studies [62,63] found that some species (e.g., waterbirds in Spain [27] may choose dams as a permanent source of water and habitat, over waterbodies with fluctuating hydroperiods, such as wetlands, especially during long hot and/or dry periods [63–65]. However, the increase in exotic and predatory species in dams with permanent water poses a challenge to biodiversity [65]. Further research on effective management strategies is essential, laying the foundation for a comprehensive exploration of farm dam management in support of biodiversity conservation.

The biodiversity values of farm dams are dependent on how dams are managed. However, most farm dams are constructed and maintained for economic purposes rather than for conservation or aesthetics [13]. Older dams that are constructed to be deep and steep-sided go through succession, changing these attributes over time creating shallows with littoral zones that can act as habitat for numerous organisms. However periodic dredging, a common management practice in older dams, returns the dams to their original state [57], greatly reducing the habitat value of these waterbodies [66]. Recent work by Westgate et al. [65] has shown that management practices such as fencing farms dams to limit direct livestock access increases aquatic and riparian vegetation cover, which is likely to have flow-on benefits for biodiversity conservation. Simple steps such as increasing habitat complexity and heterogeneity within dams, and limiting disturbance, have all been identified as important for farm dam ecosystems [67]. Buffers between dams and farmed areas, such as vegetation strips [68], hedges [69], and riparian strips [70] can help mitigate agricultural impacts on dams, by filtering pollutants, providing corridors between habitats, and reducing stock access. Additionally, buffers can be designed to direct ecosystem services from farm dams to agricultural lands, such as filtering water runoff to improve soil fertility and providing habitat for pollinators to enhance crop productivity. There has also been success in countries such as Australia [71] and New Zealand [72], with programs that reward landholders for making biodiversity friendly farming decisions such as the ones described above. These programs could be tailored specifically for farm dams. Such programs may lead to partnerships among multiple stakeholders in agroecosystems–farmers, government, practitioners, scientists, NGOs, and the wider population [72].

Our results show that farm dams can have a positive influence on biodiversity in landscapes where loss and degradation of natural waterbodies has occurred. However, there are limits to the biodiversity value of farm dams [73,74], and their establishment must consider their contribution to biodiversity holistically, taking into account the wider landscape, including the potential impact on or loss of very small wetlands, hydrological regimes, migration patterns [26], and the spread of pests and disease [72]. It is imperative to recognize that while farm dams can potentially provide habitat for biodiversity, their creation and primary purpose is typically for agricultural use rather than biodiversity enhancement and the preservation of natural wetlands should take precedence over the construction of farm dams. The biodiversity values associated with farm dams often emerge as by-products of their construction and management practices. Nevertheless, a farm dam that is well managed in conjunction with effectively conserved natural waterbodies can be an important contribution to the biodiversity of a landscape [61].

### 4.2. Key knowledge gaps

While research on the biodiversity value of farm dams has increased, significant knowledge gaps persist. Despite extensive study on the impact of agricultural intensification on farmland biodiversity, little attention has been given to its potential effects on dams in agricultural landscapes. We anticipate that intensified land use in farm dam catchments may lead to reduced species richness and habitat quality [75]. Furthermore, the significance of farm dams in supporting biodiversity may be more pronounced in areas with limited alternative habitats and prevalent land use intensification [76–78]. However, detailed comparisons between farm dams and natural wetlands are scarce, comprising less than 10% of the literature. Additionally, research on managing dams alongside natural wetland conservation is limited. We recommend that future research on farm dams focuses on: (1) quantifying how the physical characteristics of farm dams (e.g., size) influence variables such as species richness, (2) understanding how land use intensity in farm dam catchments influences variables such as water quality, and (3) experimentally quantifying the impact of management practices on the biodiversity values of farm dams. As farm dams are ubiquitous in many agricultural landscapes, better understanding of their biodiversity values and how these can be enhanced by management could lead to improved conservation outcomes across large areas.

## Supporting information

S1 Checklist PRISMA 2009 checklist. (DOC)

S1 Appendix Appendix 1. Categories and the corresponding variables and definitions from each study.
(TIFF)

S2 Appendix Appendix 2. Complete list of the 102 articles included in this review. Including article name, year of publication, authors and journal of publication.
(TIFF)

S3 Appendix Appendix 3. Representative summary table for the risk of bias assessment. Green cells with (plus) indicate a low risk of bias; yellow cells with (question mark) indicate an unknown risk of bias; red cells with (hyphen) indicate a high risk of bias.
(DOCX)
