# Supplementary figures and images for: The ecological and biodiversity conservation values of farm dams: A systematic review
Source: PLoS One. 2024 May 13;19(5):e0303504. doi: 10.1371/journal.pone.0303504 (PMC11090361; doi:10.1371/journal.pone.0303504)

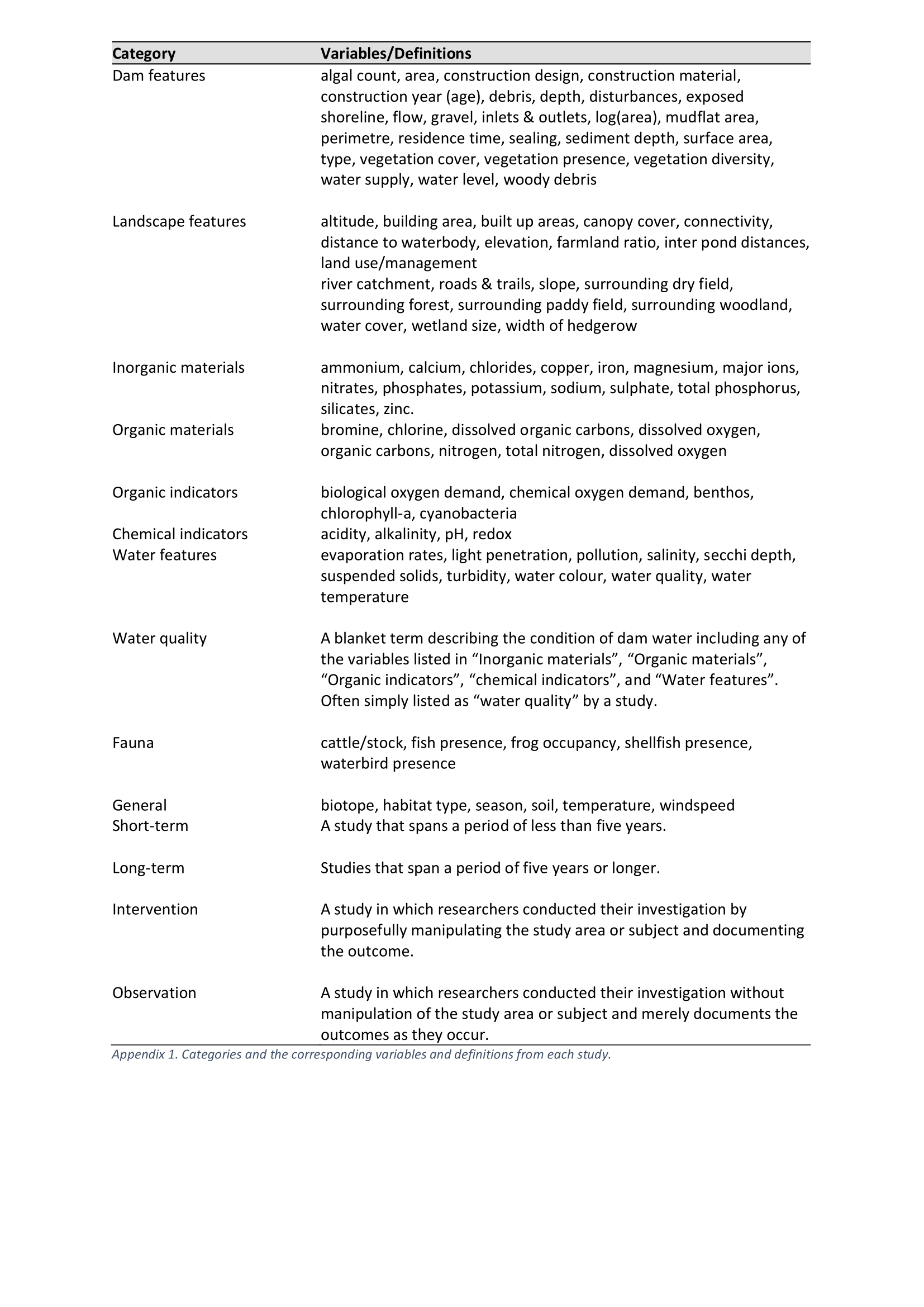

Supplement: S1 Appendix — Categories and the corresponding variables and definitions from each study. (TIFF) [file pone.0303504.s002.tiff]
