# Supplementary material for: The ecological and biodiversity conservation values of farm dams: A systematic review
Source: PLoS One. 2024 May 13;19(5):e0303504. doi: 10.1371/journal.pone.0303504 (PMC11090361; doi:10.1371/journal.pone.0303504)
Supplement: S3 Appendix — Representative summary table for the risk of bias assessment. Green cells with (plus) indicate a low risk of bias; yellow cells with (question mark) indicate an unknown risk of bias; red cells with (hyphen) indicate a high risk of bias. (DOCX) [file pone.0303504.s004.docx]

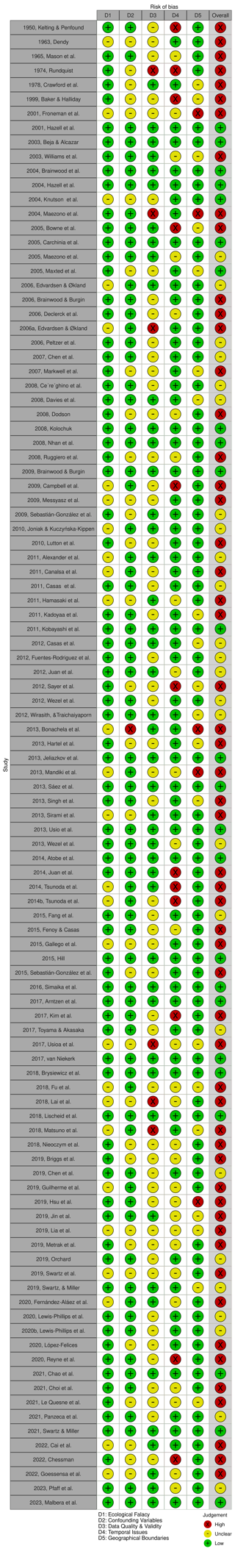


Appendix 3. Representative summary table for the risk of bias assessment. Green cells with (plus) indicate a low risk of bias; yellow cells with (question mark) indicate an unknown risk of bias; red cells with (hyphen) indicate a high risk of bias
